# The European Pediatric Surgical Audit: Improving Quality of Care in Rare Congenital Malformations

**DOI:** 10.1055/a-2551-2056

**Published:** 2025-04-15

**Authors:** Nadine M. Teunissen, Daniel Rossi, Michel W. Wouters, Simon Eaton, L.W. Ernest van Heurn, Rene Wijnen

**Affiliations:** 1 Department of Pediatric Surgery and Pediatric Intensive Care Unit, Erasmus Medical Center, Rotterdam, The Netherlands; 2 Department of Women's and Children's Health, Karolinska Institute, Stockholm, Sweden; 3 Dutch Institute of Clinical Auditing, Leiden, The Netherlands; 4 Department of Pediatric Surgery, University College London Institute of Child Health, London, United Kingdom; 5 Department of Pediatric Surgery, Emma Children's Hospital, Amsterdam UMC, University of Amsterdam and Vrije Universiteit Amsterdam, Amsterdam, The Netherlands

**Keywords:** pediatric surgery, audit, rare congenital malformations, quality of care

## Abstract

Since 2019, the European Pediatric Surgical Audit (EPSA) has been the official registry of the European Reference Network for Inherited and Congenital Anomalies (ERNICA). The primary aim of this prospective patient registry is benchmarking (quality of) care for patients with rare congenital malformations throughout Europe. Data collected comprise baseline, treatment, and outcome variables, permitting calculation of disease-specific, hospital-level quality indicator results reflecting between-hospital variation. This practice and outcome variation is fed back as actionable information to clinicians on a web-based, real-time dashboard to help focus local and central improvement initiatives. Secondly, realizing joint research initiatives with quality improvement purposes through secondary data use will increase our knowledge of these rare conditions and optimize care. Currently, 27 hospitals in 15 European countries have connected to this unique, European-wide audit. Henceforward, the focus will be on the further expansion of hospitals and diseases, as EPSA aspires to become all-encompassing, including all European patients with congenital malformations.

## Introduction

A clinical audit is a quality-of-care measurement tool offering clinicians reliable, benchmarked insight into their performance, thereby inspiring improvement initiatives. 1 This concept has proven useful in conditions such as breast cancer and colorectal cancer by decreasing practice variation between hospitals, consequently decreasing outcome variation and improving the quality of care. 2 3 4 5 6 7 Furthermore, the collected, real-world data in such a patient registry can be used for research, filling gaps in evidence or knowledge. 8 Finally, the data can be used to monitor the effectiveness of policy changes or assess outcomes in the context of costs in value-based health care. 9 These principles form the premise of the European Pediatric Surgical Audit (EPSA), established in 2014 by the Dutch Association of Pediatric Surgeons as a nationwide clinical audit covering all patients undergoing treatment for six congenital anomalies. 10

However, the ability to compare processes and outcomes of rare diseases in a small population country such as the Netherlands is limited, especially as care of some conditions has been centralized to two or three hospitals since 2017. 11 Then, the European Union funded the initiation of European Reference Networks (ERNs): virtual networks involving health care providers across Europe, facilitating discussion, and disseminating knowledge, specifically on rare diseases, including funding for developing patient registries. 12 The European Reference Network for Inherited and Congenital Anomalies (ERNICA) was launched in 2017 as part of this initiative. 13 Recognizing the importance of cross-country collaboration, EPSA and ERNICA merged efforts and resources to form the EPSA|ERNICA registry in 2019. 14

This health policy paper describes the initiation of the EPSA|ERNICA Registry (henceforth EPSA), a clinical audit to continuously monitor, compare, and improve the quality of care for patients with rare inherited and congenital anomalies across Europe. It presents its organizational structure, governance, development, progress in the last years, and the future utilization of its results. Moreover, it identifies barriers to setting up a quality control registry and describes prospects for further development.

## How to Audit: An Overview of European Pediatric Surgical Audit Development

EPSA is a prospective patient registry covering five congenital conditions requiring pediatric surgical care in infancy or early childhood: esophageal atresia, congenital diaphragmatic hernia, Hirschsprung's disease, omphalocele, and gastroschisis. The incidence of these rare anomalies ranges from 1 to 3 per 10,000 live births in Europe. 15 16 All patients receiving primary treatment for one of these conditions in the participating hospitals are eligible for inclusion in EPSA. In earlier stages, the registry also included anorectal malformations and biliary atresia; however, these conditions are now included in registries of other reference networks (eUROGEN and ERN RARE-LIVER, respectively).

### Organization and Structure of the European Pediatric Surgical Audit

EPSA is one of 26 health care professional-driven, disease-specific registries facilitated by the Dutch Institute for Clinical Auditing (DICA). 1 This well-established, non-profit institute, employing, among others, health care professionals, data analysts, methodologists, and operational staff, provides a framework incorporating fundamental legal, technical, and methodological aspects, thereby taking advantage of the ensuing economies of scale. In contrast to the other disease-specific registries hosted by DICA, EPSA is the first international audit covering multiple rare conditions. This has consequences for patient volumes, the statistical power of result analysis, and the clinical interpretation of benchmarks and deviations thereof. 17

**Fig. 1:**
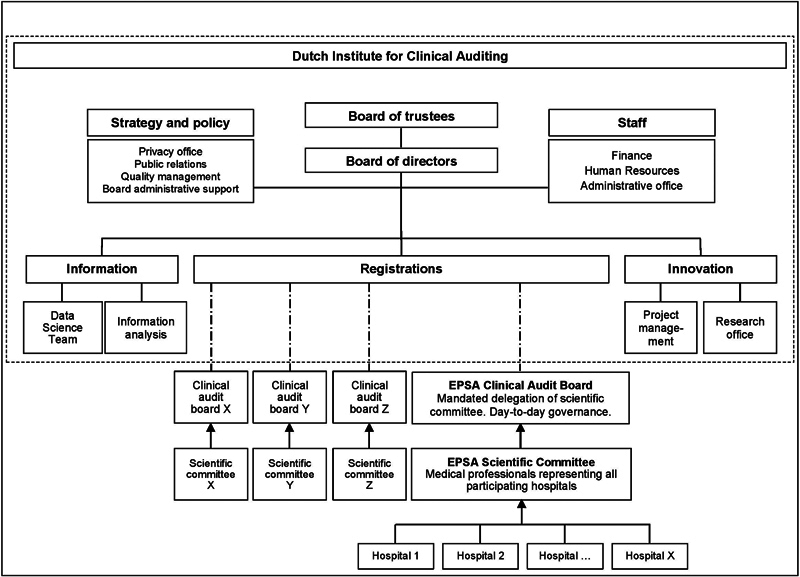
Organizational structure of the EPSA|ERNICA Registry. EPSA, European Pediatric Surgical Audit; ERNICA, European Reference Network for Inherited and Congenital Anomalies.

Supported by a registry manager, health care professionals oversee EPSA in two governing bodies: the scientific committee and the clinical audit board. The scientific committee, composed of representatives of participating hospitals, decides on the nature of the data collected, the use of data in benchmarking and quality assurance, and data access. A smaller delegation of the scientific committee, appointed and mandated by the scientific committee itself, forms the clinical audit board. Together with the registry manager, this clinical audit board is responsible for the operational, day-to-day implementation of the scientific committee's decisions. Figure 1 depicts the organizational and governance structure of DICA and EPSA.

### Participating Hospitals

Clinical auditing and quality assurance registries initiated by medical professional associations are widely accepted as common practice in the Netherlands for many surgical conditions. Following this movement, in 2014, the Dutch Association of Pediatric Surgeons initiated EPSA, with mandatory participation for all six pediatric surgical units in the Netherlands. After receiving the first ERN grant in 2020, the expansion of EPSA started by inviting ERNICA expert centers to participate. Later, hospitals outside the ERNICA network were invited, provided they treat at least five new patients per year for one of the conditions in the registry. Naturally, General Data Protection Regulation (GDPR)-adherent contracts are in place with each hospital, legitimizing data collection for quality control. Each hospital usually goes through several reviewing and adaptation rounds before signing these contracts to fit local legislation and the hospital's interpretation. EPSA is financially supported through ERNICA funding as granted by the European Commission in the Third Health Program, HP-PJ-2019 and the Fourth Health Program, EU4H-2022-ERN-IBA. Consequently, hospitals do not pay fees for their subscription to EPSA nor the maintenance of the registry platform. However, administrative costs, such as data entry costs, are not reimbursed.

### Data Entry, Data Processing, and Data Quality

As a data controller, the hospital is responsible for the prospective data entry and the accuracy thereof. Data are manually entered into a secured, web-based system, commonly by one of the treating medical specialists, but also by trained researchers, nurses, or data managers. The collected variables entail general patient and prenatal characteristics—similar for all patients regardless of their condition—in addition to disease-specific characteristics and process variables—mainly concerning primary treatment and outcomes. After 1 year, variables measuring 1-year outcomes must be added to complete the patients' data entry definitively. If a patient has multiple conditions, each condition is registered separately.

The most recent versions of each disease-specific case report form are available online. 18 The number of variables entered per patient ranges from 81 to 146 for the different diseases. However, most of these variables are requested conditionally, only appearing if previous responses indicate they are applicable, limiting the administrative burden of answering irrelevant questions. Depending on the disease and the complexity of the patient's history, it takes approximately 15 to 30 minutes to register each patient. Several validation measures are built into the survey program to ensure data quality upon entry. If data entered are unlikely to occur, the registrar receives an immediate error report, for example, because a sequence of dates is improbable or if data are partially skipped.

After entry, data are processed, pseudonymized, and safely stored by Medical Research Data Management (MRDM), a trusted NEN7510- and ISO27001-certified third party, in adherence to the GDPR and local regulations. Due to differences in local privacy legislation and the individual hospital's interpretation, some hospitals pseudonymize their data before data entry, not affecting the following standard processing method.

Generally, the registry dataset is reviewed and adapted in a standardized annual cycle. Each year, adaptations proposed by registering professionals are discussed in the ERNICA disease-specific, expert work streams and, if supported, implemented in the dataset. In addition to this yearly updating process, an extensive revision of the current dataset is ongoing, including a quality indicator selection Delphi process for each condition, which is explained in more detail in the next paragraph. The final disease-specific datasets will include the variables necessary to measure process (i) and outcome (ii) quality indicators and the patient and disease characteristics to adjust for when comparing these outcomes between institutions or countries (iii). The operational usage of these variables, including definitions and values, will be based on literature, operational usage in similar patient registries, and expert opinion. The resulting uniformity will increase interpretability, comparability, and interoperability, as is desirable when using the findable, accessible, interoperable, and reusable (FAIR) principles for scientific data. 19

### Measuring Quality: Quality Indicators

Quality indicators are critical for measuring (quality of) care and returning useful benchmarked information. Indicators can be categorized into structure, process, and outcome, each measuring other aspects of the care pathway. 20 Although outcome indicators such as survival rate are concrete, clear endpoints, these often reflect multiple underlying causes of variation. Additionally, the time needed to pass before resulting in useful information, especially in rare diseases with low patient numbers and uncommon adverse outcomes, limits its functionality as an actionable foundation for improvement initiatives. 21 Process indicators, such as the time between diagnosis and surgery or the percentage of patients undergoing a particular diagnostic test, might be convenient, more sensitive alternatives to these outcome indicators. However, for rare EPSA conditions, a link between the concerning aspect of the care process and the desired outcome often has not (yet) been scientifically established. 17 Hence, the optimal quality indicator set incorporates both process and outcome indicators. Lastly, measuring structural indicators might be valuable, such as a hospital's number of patients treated yearly for a specific condition or the availability of certain imaging techniques. These indicators reflect essential differences in care when structural health care aspects vary across benchmarked institutions, although they generally are more static and less prone to change.

In addition to comprising both process and outcome indicators, the quality indicators must reflect the consensus of all stakeholders, importantly including patient groups as well as health care professionals, on what entails good (quality of) care. 5 Moreover, the implemented quality indicator set should apply to all participating countries' health care practices and be selected methodologically to ensure confidence in EPSA as a quality measurement tool and attach weight to its results. The quality indicators currently used are based on the consensus of Dutch pediatric surgeons in 2015. 10 After a literature review, we developed a new method for quality indicator selection, including a systematic literature review and review of prevailing guidelines, followed by a three-round Delphi questionnaire among European health care professionals—of all involved disciplines—and patient representatives. This method was successfully pilot tested for esophageal atresia, after which minor amendments were made to the protocol regarding the extent of the (systematic) literature review, the Delphi timeline, and the number of items on the long list of indicators presented to the panels during this Delphi. 22 According to this revised protocol, the indicator sets for other conditions are currently being developed. Researchers and clinicians in the extended ERNICA network lead these projects.

### Accomplish Improvement

The insights gained from this registry should be systematically utilized to maximize the effectiveness of EPSA as a quality-of-care measurement tool. First and foremost, a high-quality feedback system is crucial, directly providing health care professionals with insight into their performance. For that purpose, DICA developed the Codman Dashboard. This weekly updated dashboard shows the hospital's performance compared to other anonymized hospitals in funnel plots with 95% confidence intervals around the benchmark. It also generates lists of patients meeting the criteria for specific quality indicators, for example, those who developed a specific complication. These lists allow participating professionals to analyze the disease course, treatment, and actions that led to desirable or unwanted events in a specific patient group.

The interactive, disease-specific annual feedback session, held live during the annual ERNICA meeting, is another example of an improvement initiative organized centrally. Depth analysis of a specific topic is prepared and discussed by all participating health care professionals. The findings and conclusions are then reported back to all participants. With accumulating numbers and periodic revisiting of these specific topics, we hope to recognize trends and see care improvement in the future.

Next to these centrally organized initiatives, local improvement initiatives have emerged. For example, some hospitals use EPSA data and the Codman dashboard as the basis for routine complication reviews or for discussing other outcomes, thereby continuously identifying areas requiring additional attention. Other hospitals use the data to assess the care pathway or for educational purposes, examining the data with staff members, residents, and interns.

### Ethical Considerations and Data Access

To fairly evaluate hospital variation, complete patient population coverage to ascertain comparable patient cohorts is essential. Using informed consent would inherently introduce bias. EPSA data collection for quality assurance is thus principally based on an opt-out system rather than informed consent, legally relying on Article 9, paragraph 2, sub I of the General Data Protection Regulation. 23 If hospitals deemed informed consent non-negotiable, we permitted the implementation thereof locally to make EPSA as accessible as possible. These hospitals provide EPSA yearly with the number of patients that denied their inclusion, thereby limiting the ensuing bias as much as possible and increasing the interpretability of their hospital's results.

Reusing collected quality control data of this large, international cohort of patients with rare conditions might generate new insights and knowledge that would have been difficult to obtain in the real world. Therefore, secondary use of this valuable data is enabled, albeit under strict conditions regulated in the governance protocol of EPSA. Only pseudonymized, non-retraceable data can be requested through an extensive research application process with an assessment of relevance, methodology, and data availability by EPSA's data access committee. Under Dutch law, a separate ethical board review of research applications is not required; however, individual hospitals might still request local ethics approval to concur with local legislation. At least two centers must participate in each research application to encourage hospital collaboration. Lastly, all participating hospitals are recognized for contributing their data by offering collaborative authorship, meeting the requirements of scientific journals.

## Early Registry Results: Where Are We Now?

**Fig. 2:**
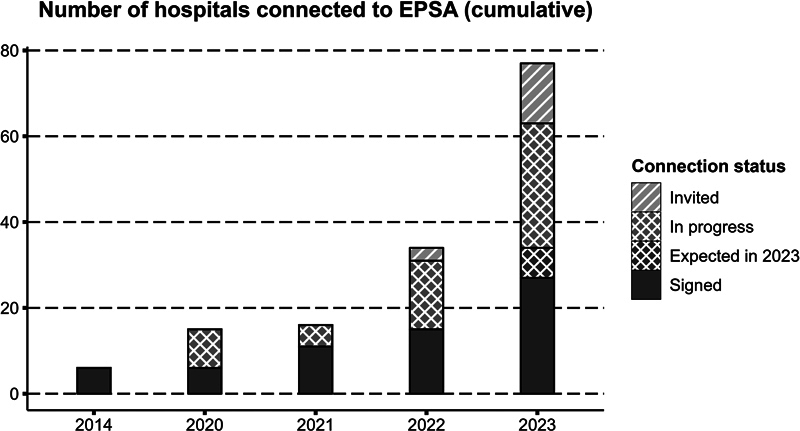
Number of hospitals connected to EPSA. The numbers for 2023 are preliminary, as expansion is ongoing. EPSA, European Pediatric Surgical Audit.

In 2014, EPSA was initiated by the six hospitals with a pediatric surgical unit responsible for treating Dutch patients with a condition registered in EPSA. After the international expansion kick-off and ERNICA expert centers' first invitations in 2020, this number grew to 15 hospitals in 2022. An overview of participating hospitals is depicted in Fig. 2 . In December 2023, 11 additional hospitals joined, and 8 are expected to join during the next year. Another large group is currently in the earlier stages of connecting (EPSA|ERNICA Registry Group).

### Increased Registration

**Fig. 3:**
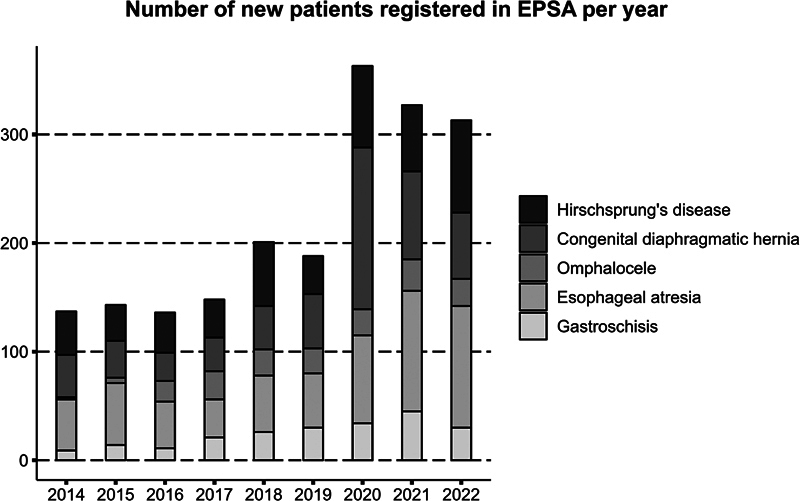
Number of new patients with esophageal atresia, Hirschsprung's disease, omphalocele, gastroschisis, or congenital diaphragmatic hernia, registered in EPSA per year. The numbers for 2022 are preliminary, as registration is ongoing. EPSA, European Pediatric Surgical Audit.

**Fig. 4:**
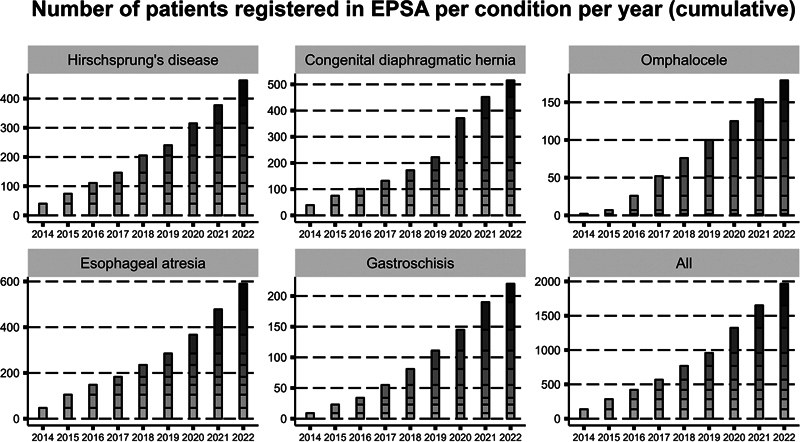
Cumulative number of patients registered in EPSA per condition. The numbers for 2022 are preliminary, as registration is ongoing. EPSA, European Pediatric Surgical Audit.

Until 2020, the six Dutch hospitals registered 1,144 unique patients with one or more of the five EPSA conditions. A manual data verification process to assess data quality is ongoing for Dutch data registered until 2021. As EPSA expanded, in parallel, the number of registered patients increased. In December 2023, 2,166 unique patients had been registered in EPSA, in addition to 634 patients with an anorectal malformation that are only registered in the Netherlands. Figure 3 displays the number of patients registered annually for the five conditions in Europe, in which numbers for 2022 are still incomplete because of ongoing registration. Figure 4 illustrates the cumulative number of patients in EPSA per condition. As the number of participating hospitals grows exponentially, so will the number of registered patients during the coming years.

## Learn… and Improve! Future Perspectives of European Pediatric Surgical Audit

This paper describes the initiation, design, and further development of the EPSA. This clinical audit, a registry of patients with rare inherited and congenital anomalies by clinicians for clinicians, aims to improve (quality of) care for these patients by measuring, comparing, and evaluating variation between hospitals, regions, and even countries. To that end, the methodologically selected structure, process, and outcome indicators involving all stakeholders are systematically calculated and fed back as benchmarked information to the health care professionals. This insight then enables them to recognize opportunities for improvement. The number of European hospitals participating in the audit grows steadily and, subsequently, the number of patients registered in EPSA.

### Improving the Improvement Cycle

To improve (quality of) care, gained insight through auditing should be translated to tailored change strategies and interventions. 24 Examples of such interventions might include (peer) visitation, adding annual targets to quality indicators, or publicly disseminating quality indicator results. These are further detailed in Supplementary Material S1 (available in the online version only). The sine qua non is the commitment of health care professionals and participating centers to invest resources in the registry and self-direct and sustain improvement efforts. 25 This commitment should be encouraged and supported by ERNICA. 4 6 In addition, ERNICA collecting, reviewing, and disseminating successful improvement initiatives will allow for continuous optimization of the improvement cycle itself. Finally, disease-specific studies using EPSA data should follow to assess this anticipated impact of EPSA on the quality of pediatric surgical care in Europe, for example, by examining trends over time in variation. 3

### Optimizing Measurement and Feedback

EPSA-generated reports on variation must be relevant, valid, reliable, and recognized as such to be used in clinical practice. 26 Therefore, quality indicators should be consensus-based and regularly reviewed to assess continued applicability, feasibility, and discriminatory power, adapting or discontinuing indicators if necessary. Correcting for case-mix should eventually be enabled to assure the validity of measured (quality of) care, ensuring that measured variation genuinely reflects between-hospital variation rather than differences in the underlying patient population or random variation. 17 26 However, these models require high patient volumes, and the rare nature of the included conditions and the related infrequent occurrence of specific outcomes limit statistical power. 17 Thus, these models and their value are yet to be established.

Presenting the quality indicator results in a clear, easy-to-interpret way is equally critical for a healthy clinical audit. Funnel plots with a 95% confidence interval, as used in the DICA-developed Codman Dashboard, effectively visualize between-hospital outcome variation, with the ability to identify outliers around the benchmark. 27 However, again, the rarity of the conditions affects the power of these analyses and limits the ability to detect outliers. A more useful Codman function in rare diseases may be a casewise analysis of transpired treatment and its outcomes through generating specific patient lists, thereby identifying focus points for improvement initiatives. This approach has proven useful in other improvement initiatives. 28 29

### Overcoming Obstacles to Achieve Effective and Sustainable Auditing

The effect of auditing and feedback is not undisputed. Other audits hosted by DICA have decreased between-hospital variation, thereby increasing the overall quality of care. 1 7 30 However, a 2006 systematic review showed mixed results, identifying studies that did not show an association between audit and improved (quality of) care. 31 Nevertheless, they argue that auditing effects may be more prominent when health care professionals are formally held responsible for acting on the feedback, circling back to the importance of the participants' commitment, as mentioned above. A 2012 revision of this Cochrane review confirms auditing and feedback as beneficial tools, mainly depending on the form of provided feedback and the baseline performance. 32

Throughout the ongoing international expansion of EPSA, several obstacles to effective and sustainable auditing were identified. First, although the concept of clinical auditing is well-established in the Netherlands, clinicians, hospitals, and their legal teams are unfamiliar with it in other countries. Furthermore, despite the overarching GDPR that is in effect in all participating countries, countries' legislation and interpretation differences complicate the legal framework. The division in using informed consent is a culmination thereof. In the Netherlands, new, more explicit legislation on pseudonymized data collection in quality control registries is being implemented, addressing issues such as data controllership and the exemption of informed consent. In the future, the successful implementation of this legislation in the Netherlands might serve as an example to other countries and Europe as a whole. Enabling clinical audits across borders, not only for rare diseases but for all conditions, may improve the entirety of the quality of care in Europe and expand our general evidence-based knowledge, benefiting a vast group of patients.

The administrative burden of registries is a second barrier to sustainable data collection. The balance between gaining useful clinical information, the perceived benefits for the hospitals and public interest, and the time spent on data entry is crucial. Ideally, data would be automatically extracted from electronic patient records. However, achieving this level of interoperability is a big task, requiring revision of the various electronic patient file systems and health data registration methods. The digitization of society, combined with increased recognition of the importance of reusable data as evidenced by the weight attributed to FAIR principles across the entire health data landscape, shall undoubtedly accelerate this process. 19 EPSA will use the coming years to FAIRify all data points to facilitate easy extractability and explore the costs and benefits of automated data extraction.

Another challenge is safeguarding high data quality, which could arguably be considered the fifth FAIR principle. After all, low quality renders even FAIR data useless. 33 The data entry program contains several automated error checks to prevent incorrect data from being entered. However, once entered, verification of this registered data is complicated. Manual verification, thus comparison of registered data with the original data source by a third party, is complicated by language barriers in an international context. Therefore, a practical, feasible international data verification method should be researched and implemented in the coming years, introducing an opportunity for collaboration with registries of other ERNs.

Finally, there is the problem of competing registries. For example, France has a mandatory registry for esophageal atresia and a national, administrative, and epidemiological rare disease register, so French centers, for now, have decided against joining the EPSA registry. 34 35 Other registries, such as the long-established congenital diaphragmatic hernia study group, are also active, increasing the administrative burden where centers wish to contribute to EPSA and another registry. 36 Future work will be necessary to align data dictionaries between these registries.

### The Future of European Pediatric Surgical Audit

During the coming years, we want to accomplish several goals. First, we aim to expand the number of conditions included in the EPSA registry. Creating a singular platform for most treated conditions increases the accessibility and feasibility of registration for the involved health care professionals. Other intended dataset additions are disease-specific modules extending past the currently limited follow-up, measuring long-term care and outcomes. Again, the benefit of this extra data retrieval should outweigh the corresponding increase in administrative burden. There is also increasing interest in measuring patient-reported outcome measures, for example, through questionnaires on quality of life and the linkage thereof to the collected clinical data, for which possibilities will be explored.

This study describes the introduction of a clinical audit for rare congenital pediatric conditions in Europe. Although there are differences between national health care systems within Europe, the overall health care landscape is reasonably comparable (see Supplementary Material S2 [available in the online version only] for more information), as opposed to the health care landscape of, for example, the United States. Clinical auditing in the United States' more fragmented, private health care sector might be more complicated, as other interests—such as from the individual clinics or physicians—are at stake. 4 Moreover, differences in legislation complicate data processing and data transfer between the EU and other countries. However, we believe global collaboration benefits all patients with rare conditions; thus, we maintain contact with other patient registries on the conditions included in EPSA, other reference network registries, and institutions such as the European Joint Programme on Rare Diseases (EJPRD), continuously exploring ways to cooperate.

Quality measurement might be even more effective when comparing expert and non-expert centers. Therefore, the final focus will be connecting most European centers treating these conditions. Presently, participation in EPSA is voluntary, which in clinical auditing may lead to the underrepresentation of underperforming hospitals. 5 In the future, connecting to EPSA could be further encouraged by making participation a clinical performance indicator or even mandatory to obtain recognition as an ERNICA expert.

## Conclusion

The EPSA is a unique quality measurement tool currently being widely implemented across Europe. The innovative platform enables pediatric professionals to gain insight into treatment variations in rare congenital anomalies and their performance. By recognizing between-hospital variation in treatment and outcome and subsequently focusing quality improvement efforts, this audit can elevate the entire pediatric (surgical) care to a higher level. Cooperation within and between centers, across specialisms and borders, is essential to achieve this goal. Ultimately, EPSA aspires to become an all-encompassing audit, including all European patients with various (congenital) pediatric malformations. The EPSA|ERNICA Registry team will work diligently and persistently to achieve these goals in the coming years.
