# Supplementary material for: The European Pediatric Surgical Audit: Improving Quality of Care in Rare Congenital Malformations
Source: Eur J Pediatr Surg. 2025 Apr 15;35(5):426–34. doi: 10.1055/a-2551-2056 (PMC12413265; doi:10.1055/a-2551-2056)
Supplement: Supplementary file 1 — Supplementary Material [file 10-1055-a-2551-2056-s2023126822oa.pdf]

EPSA|ERNICA Registry Group—Representatives of All Hospitals Participating in the European Surgical Audit, Contributing to Governance, Maintenance, and Further Development of the Audit

1. Prof. R.M.H. (René) Wijnen—Pediatric surgeon
   Erasmus University Medical Centre – Sophia Children's Hospital, Rotterdam, The Netherlands
2. Prof. L.W.E. (Ernest) van Heurn—Pediatric surgeon
   Amsterdam UMC – Emma Children's Hospital, University of Amsterdam, Amsterdam, The Netherlands
3. Prof. J.B.F. (Jan) Hulscher—Pediatric surgeon
   University Medical Center Groningen – Beatrix Children's Hospital, University of Groningen, Groningen, The Netherlands
4. Dr. M.Y.A. (Maud) Lindeboom—Pediatric Surgeon
   University Medical Center Utrecht, Utrecht, The Netherlands
5. Prof. I. (Ivo) de Blaauw—Pediatric surgeon
   Radboud University Medical Center, Nijmegen, The Netherlands
6. Prof. W.G. (Wim) van Gemert—Pediatric surgeon
   Maastricht University Medical Centre, Maastricht, the Netherlands
7. Dr. S. (Simon) Eaton—Developmental biologist
   UCL GOS Institute of Child Health, London, United Kingdom
8. Dr. R. (Rony) Sfeir—Pediatric Surgeon
   Centre Hospitalier Universitaire de Lille, Lille, France
9. Prof. T. (Tomas) Wester—Pediatric surgeon
   Karolinska University Hospital, Stockholm, Sweden
10. Prof. M.P. (Mikko) Pakarinen—Pediatric surgeon
    Children's Hospital, Helsinki University Central Hospital, Helsinki, Finland
11. Prof. J. (Jorgen) Thorup—Pediatric surgeon
    Rigshospitalet, Copenhagen, Denmark
12. Dr. J. (Julia) Brendel—Resident Pediatric Surgeon
    Hannover Medical School, Hannover, Germany
13. Dr. K.B. (Katrin) Zahn—Pediatric surgeon
    Universitätsklinikum Mannheim, Mannheim Germany
14. Prof. K. (Kristin) Björnland—Pediatric surgeon
    Oslo University Hospital, Oslo, Norway
15. Dr. P. (Pernilla) Stenström—Pediatric surgeon
    Skåne University Hospital Lund, Lund, Sweden
16. Dr. M. (Marta) Erculiani—Pediatric surgeon
    Azienda Ospedaliera SS, Antonio e Biagio e Cesare Arrigo, Alessandria, Italy
17. Prof. Dr. M. (Marc) Miserez—Abdominal surgeon
    University Hospitals Leuven, Leuven, Belgium
18. Dr. R. (Rebeka) Pechanová—Resident Pediatric Surgeon
    University Hospital Bratislava, Bratislava, Slovakia
19. Dr. A. (Andrea) Conforti—Pediatric surgeon
    Ospedale Pediatrico Bambino Gesù, Rome, Italy
20. Assoc. Prof. V. (Vlad) David—Pediatric surgeon
    "Victor Babes" University of Medicine and Pharmacy – "Louis Turcanu" Emergency Children's Hospital, Timisoara, Romania
21. Dr. A. (Antonio) di Cesare—Pediatric surgeon
    Fondazione IRCCS Ca' Granda Ospedale Maggiore Policlinico, Milan, Italy
22. Dr. Z. (Zane) Ābola—Pediatric surgeon
    Children's Clinical University Hospital, Riga, Latvia
23. Dr. R (Riccardo) Guanà—Pediatric surgeon
    Regina Margherita Children's Hospital, Turin, Italy
24. Dr. N. (Nadezhda) Tolekova—Pediatric surgeon
    "N.I. Pirogov" University Hospital, Sofia, Bulgaria
25. Dr. M. (Maria) Stockinger—Pediatric surgeon
    Kepler Universitatsklinikum, Linz, Austria
26. Dr. L. (Leopoldo) Martinez—Pediatric surgeon
    Hospital Infantil La Paz, Madrid, Spain
27. Dr. J. (Jordi) Prat—Pediatric surgeon
    Sant Joan de Déu Barcelona Children's Hospital, Barcelona, Spain
28. Dr. L. (Lucas) Matthyssens—Pediatric surgeon
    Ghent University Hospital, Ghent, Belgium
29. Dr. S. (Sanja) Sindjic-Antunovic—Pediatric surgeon
    University Children's Hospital Belgrade, Belgrade, Serbia

Supplementary Material S1: Potential Change Strategies in Health care

Much research has been dedicated to identifying effective change strategies. The development of dashboards containing computer-supported feedback messages based on the receiving party and the desired effect is an effective example.[1] Another possible intervention to accomplish change is a site visitation of an independent committee to hospitals performing below the benchmark to recognize strengths and weaknesses in the provided care and generate valuable priorities for change. In that respect, van den Hombergh et al. found peer visitation more effective than visitation by non-physician observers.[2] Also, annual targets could be added to quality indicators, thereby attributing a layer of direction and aspiration to the benchmarked information.[3] A last change strategy, implemented in many DICA (Dutch Institute for Clinical Auditing) patient registries, is transparency: making quality indicator results of each participating hospital publicly available. Larsson et al. found a drastic change in guideline adherence after the public disclosure of specific individual hospitals' results, decisively decreasing between-hospital variation.[4,5] The information could also be of value for patients, informing them of differences in treatment and outcome between health care providers in their region.[6] However, one should exercise caution in publishing quality indicator results in the context of rare diseases. Because of low patient volumes and low prevalence of outcome events, identified treatment or outcome variation may rely on coincidence. Hence, such data often does not warrant unambiguous conclusions and might be wrongfully interpreted by non-professionals. A first step in publishing hospital variation could be the publication of data quality results, such as disease-specific data completeness, which may also encourage health care professionals to register their patients timely and accurately.

Supplementary Material S2: European Health care Landscape

Although supplemented by the European Union (EU) health policies and actions in public health, the responsibility for public health and medical care lies with the member states of the EU.[7] Therefore, within Europe, health care systems differ. Many (cluster) analyses describing these differences in European national health care systems are available, such as Wendt's extensive review of health care systems in 2009.[8] An example of variation lies in the regulation of health care insurance, which might be based on government funding or privatized with specific regulations. Similarly, health care providers might be public or private. In some countries, such as the Netherlands and Sweden, pediatric surgical care is centralized and concentrated in university hospitals.[9,10] Other countries, such as Germany, have a more decentralized system, although some form of centralization in treating these conditions has also occurred there.[11,12] However, underlying principles in all EU member states, that is, access to basic health care for all, including care for these congenital conditions, are similar in all these countries, legitimizing cross-border registration and comparison, especially in these rare conditions.

References

1 Landis-Lewis Z, Brehaut JC, Hochheiser H, Douglas GP, Jacobson RS. Computer-supported feedback message tailoring: theory-informed adaptation of clinical audit and feedback for learning and behavior change. Implement Sci 2015;10(01):12

2 van den Hombergh P, Grol R, van den Hoogen HJ, van den Bosch WJ. Practice visits as a tool in quality improvement: mutual visits and feedback by peers compared with visits and feedback by non-physician observers. Qual Health Care 1999;8(03):161–166. Accessed March 11, 2025 at: http://www.ncbi.nlm.nih.gov/pubmed/10847872

3 Thorstenson A, Harmenberg U, Lindblad P, Ljungberg B, Lundstam SSwedish Kidney Cancer Quality Register Group. Impact of quality indicators on adherence to National and European guidelines for renal cell carcinoma. Scand J Urol 2016;50(01):2–8. Accessed March 11, 2025 at: http://www.ncbi.nlm.nih.gov/pubmed/26202573

4 Larsson S, Lawyer P, Garellick G, Lindahl B, Lundström M. Use of 13 disease registries in 5 countries demonstrates the potential to use outcome data to improve health care's value. Health Aff (Millwood) 2012;31(01):220–227. Accessed March 11, 2025 at: http://www.healthaffairs.org/doi/10.1377/hlthaff.2011.0762

5 Bilimoria KY. Facilitating Quality Improvement: Pushing the Pendulum Back Toward Process Measures. JAMA 2015;314(13): 1333–1334. Accessed March 11, 2025 at: http://jama.jamanetwork.com/article.aspx?doi=10.1001/jama.2015.12470

6 Damman OC, De Jong A, Hibbard JH, Timmermans DRM. Making comparative performance information more comprehensible: an experimental evaluation of the impact of formats on consumer understanding. BMJ Qual Saf 2016;25(11):860–869. Accessed March 11, 2025 at: https://qualitysafety.bmj.com/lookup/doi/10.1136/bmjqs-2015-004120

7 European Commission. EU Health Policy. 2023. Accessed January 16, 2023 at: https://health.ec.europa.eu/eu-health-policy/overview_en

8 Wendt C. Mapping European healthcare systems: a comparative analysis of financing, service provision and access to healthcare. J Eur Soc Policy 2009;19(05):432–445. Accessed March 11, 2025 at: http://journals.sagepub.com/doi/10.1177/0958928709344247

9 Wijnen MH, Hulscher JB. Centralization of pediatric surgical care in the Netherlands: Lessons learned. J Pediatr Surg 2022;57(02): 178–181. Accessed March 11, 2025 at: https://linkinghub.elsevier.com/retrieve/pii/S002234682100734X

10 Pakarinen M, Bjørland K, Qvist N, Wester T. Centralized pediatric surgery in the Nordic countries: A role model for Europe? Eur J Pediatr Surg 2017;27(05):395–398. Accessed March 11, 2025 at: http://www.thieme-connect.de/DOI/DOI?10.1055/s-0037-1606635

11 Schmedding A, Rolle U. Decentralized rather than centralized pediatric surgery care in Germany. Eur J Pediatr Surg 2017;27 (05):399–406. Accessed March 11, 2025 at: http://www.thieme-connect.de/DOI/DOI?10.1055/s-0037-1607026

12 Lacher M, Barthlen W, Eckoldt F, et al. Operative volume of newborn surgery in German University Hospitals: High volume versus low volume centers. Eur J Pediatr Surg 2022;32(05): 391–398. Accessed March 11, 2025 at: http://www.thieme-connect.de/DOI/DOI?10.1055/s-0041-1740479

 © 2025. The Author(s).
